# Increase in Bacterial Colony Formation from a Permafrost Ice Wedge Dosed with a *Tomitella biformata* Recombinant Resuscitation-Promoting Factor Protein

**DOI:** 10.1264/jsme2.ME14119

**Published:** 2015-04-04

**Authors:** Indun Dewi Puspita, Wataru Kitagawa, Yoichi Kamagata, Michiko Tanaka, Cindy H. Nakatsu

**Affiliations:** 1 Graduate School of Agriculture, Hokkaido UniversityN9 W9, Kita-ku, Sapporo, Hokkaido 060–8589Japan; 2 Bioproduction Research Institute, National Institute of Advanced Industrial Science and Technology (AIST)2–17 Tsukisamu-Higashi, Toyohira, Sapporo, Hokkaido 062–8517Japan; 3 Department of Agronomy, Purdue UniversityWest Lafayette, Indiana 47907USA

**Keywords:** Rpf, *Rhodococcus*, dormant cells, cultivation

## Abstract

Resuscitation-promoting factor (Rpf) is a protein that has been found in a number of different *Actinobacteria* species and has been shown to promote the growth of active cells and resuscitate dormant (non-dividing) cells. We previously reported the biological activity of an Rpf protein in *Tomitella biformata* AHU 1821^T^, an *Actinobacteria* isolated from a permafrost ice wedge. This protein is excreted outside the cell; however, few studies have investigated its contribution in environmental samples to the growth or resuscitation of bacteria other than the original host. Therefore, the aim of the present study was to determine whether Rpf from *T. biformata* impacted the cultivation of other bacteria from the permafrost ice wedge from which it was originally isolated. All experiments used recombinant Rpf proteins produced using a *Rhodococcus erythropolis* expression system. Dilutions of melted surface sterilized ice wedge samples mixed with different doses of the purified recombinant Rpf (rRpf) protein indicated that the highest concentration tested, 1250 pM, had a significantly (*p* <0.05) higher number of CFUs on agar plates after 8 d, approximately 14-fold higher than that on control plates without rRpf. 16S rRNA gene sequences revealed that all the colonies on plates were mainly related to *Brevibacterium antiquum* strain VKM Ac-2118 (AY243344), with 98–99% sequence identity. This species is also a member of the phylum *Actinobacteria* and was originally isolated from Siberian permafrost sediments. The results of the present study demonstrated that rRpf not only promoted the growth of *T. biformata* from which it was isolated, but also enhanced colony formation by another *Actinobacteria* in an environmental sample.

Although bacteria in the environment are exposed to various conditions that are unfavorable for their growth (25), they still survive under some very extreme settings, such as starvation, limited oxygen (27), and low temperatures (14, 31). A number of strategies are used by bacteria to survive under such stressful conditions and resume growth once conditions become more favorable (15, 29). One strategy is to enter a dormant state (22). Dormant cells remain viable, but cannot be cultivated (referred to as VNC or VBNC) when plated onto growth medium in a laboratory (25) without the addition of factors to resuscitate cells or promote growth (22). One of these factors is a secreted extracellular protein called resuscitation-promoting factor (Rpf), which has been shown to resuscitate and promote the growth of *Actinobacteria* (8, 16, 28) by cleaving cell wall components to produce peptidoglycan fragments that are thought to serve as signaling molecules for growth initiation (9). The activity of Rpf from *Actinobacteria* has been verified using purified Rpf proteins from *Micrococcus luteus* (16), *Mycobacterium tuberculosis (*18), and *Corynebacterium glutamicum* (6). Although Rpf proteins also exhibit cross species activity (16, 18, 26), its benefit to the host for promoting the growth or resuscitation of other species currently remains unknown.

We previously reported the presence of living bacteria preserved in 25,000-year-old permafrost ice wedge samples collected from the Fox tunnel in Alaska, USA (11). A number of *Actinobacteria* were grown from these samples including a new genus and species, *Tomitella biformata* AHU 1821^T (^=DSM 45403^T^=NBRC 106253^T^) (12). We demonstrated that the Rpf from *T. biformata* formed a monophyletic clade that was separate from other Rpf proteins (23). The Rpf protein obtained by cloning and expressing the *rpf* gene from *T. biformata* in the *Escherichia coli* TOP10 system displayed growth promotion and cell resuscitation activities, but was required at higher concentrations than those of previously studied Rpf proteins. However, the protein extract was not pure, yielding four bands on an SDS-PAGE gel, only two of which hybridized in western blots using the anti-His antibody. Therefore, an alternative expression system is needed for the *rpf* gene from *T. biformata* in order to determine the concentration required for optimal activity.

The genome sequences of bacteria isolated from various environments, including the human microbiome, plant rhizosphere, contaminated soils, deep sea marine sediment, hot spring run-off, and sewage sludge, carry Rpf gene sequences (22, 26), suggesting that this is a common function among *Actinobacteria* in the environment. Based on these findings and also Rpf exhibiting cross species activity to some laboratory isolates, the aims of the present study were to determine whether the purified *T. biformata* Rpf protein applied to an environmental sample increased the cultivation efficiency of bacteria and also if it had cross species activity. To achieve these objectives, a permafrost ice wedge sample collected from the Fox tunnel, Alaska, USA, from which *T. biformata* was originally isolated, was used as the environmental source of bacteria. Furthermore, the *rpf* gene from *T. biformata* was cloned into an actinobacterial *Rhodococcus erythropolis* expression system (13, 20) to reduce the production of non-target proteins using an *E. coli* expression system (23).

## Materials and Methods

### Cloning and purification of the recombinant Rpf protein

The recombinant Rpf protein expressed in *R. erythropolis* (rRpf-R) was produced using the pTip expression vector (20) provided by the Bio-production Research Institute, National Institute of Advanced Industrial Science and Technology (AIST), Sapporo, Japan. The *rpf* gene homolog from *T. biformata* including its N-terminal signal peptide was inserted into the pBAD/gIII B vector to include a his-tag sequence at the C-terminal end. The modified gene was then transferred into the pTip-QC2 vector (23). The resultant plasmid, pTip-*rpf-his*, was introduced into *R. erythropolis* M1218 using electroporation as follows. To prepare electrocompetent cells, *Rhodococcus* cells were grown in 10 mL LB medium (OD600=1.0), washed twice with 2 mL of 10% glycerol, and resuspended in 1 mL of 10% glycerol. The cells were stored at −80°C until later use. One to five micrograms of DNA was mixed with 100 μL of electrocompetent cells. Electroporation was performed under high-voltage conditions (16 kV cm^−1^, 500 Ω, and a 25 μF capacitor) using a Gene Pulser Xcell (Bio-Rad). The *R. erythropolis* M1218/pTip-*rpf-his* strain was cultured at 27°C for 12 h in 1 L of W minimal medium (34) supplemented with succinate (0.2%, w/v), sucrose (0.2%, w/v), casamino acids (0.2%, w/v), and chloramphenicol (17 μg mL^−1^). Cultures were then induced using thiostrepton (Sigma, USA) (0.5 μg mL^−1^) and incubated for 5 h. The supernatant was harvested by centrifugation at 6,000×*g* for 20 min (4°C), and the resulting supernatant was passed through a 0.2-μm filter (Nalgene) and further purified using nickel affinity chromatography as described previously (23). The filtered supernatant was plated onto R2A medium to confirm that there was no cellular growth. Proteins were quantified using the Bradford method (Quick Start Bradford reagent kit, Bio-Rad, USA), their sizes were determined using SDS-polyacrylamide gel electrophoresis (PAGE), the target was confirmed using western blot hybridization with the anti-His antibody, and the N-terminal amino acid sequence was determined using the Edman degradation method by the Instrumental Analysis Division of Hokkaido University. Some rRpf was denatured by autoclaving and used as a second negative control. The activity of the purified protein was confirmed using the growth promotion and resuscitation of *T. biformata* as described previously (23). Briefly, non-dividing cells (dormant cells) for resuscitation experiments were prepared by incubating stationary phase cells under oxygen-limited conditions for 60 d. These cells were then washed and serially diluted to eliminate any remaining dividing cells. Although cells in the non-dividing state could not be cultivated on agar medium, a microscopic analysis after staining indicated that membrane integrity was maintained. The recombinant Rpf protein was diluted in R2A broth to obtain final concentrations of 0, 1.25, 12.5, 125, and 1250 pM of proteins for mixing with ice wedge dilutions, as described below. The criterion for the apparent lag phases was according to Mukamolova *et al. (*16). Growth rates were calculated from the slope of the best fitting linear regression of log-transformed exponential growth data plotted against time.

### Permafrost ice wedge sample preparation

The permafrost ice wedge sampling site and sample sterilization method were described previously (11). Briefly, the ice block from Fox tunnel, Alaska, USA was submerged in 75% (v/v) ethanol solution for 5 s and the surface was burned to destroy any contaminating bacteria. The ice was stamped onto agar plates to confirm the absence of surface contamination. The ice block was thoroughly rinsed using 0.85% (w/v) NaCl, then melted and 10-fold serially diluted in R2A broth. Melted ice dilutions (10^0^, 10^−1^, 10^−2^, 10^−3^, and 10^−4^) were mixed with each amount of the Rpf protein (0, 1.25, 12.5, 125, and 1250 pM, and 12.5 pM denatured Rpf) being tested for a total of 30 treatment combinations in triplicate. The results from the denatured proteins were not significantly different from the 0 rRpf controls and, therefore, were not shown in the Results section. Mixtures were spread onto R2A agar medium containing antimicrobials (0.05 g nystatin L^−1^ and 0.01 g cycloheximide L^−1^) to inhibit fungal growth. Plates were incubated at 15°C for 2 weeks and colony numbers were counted daily. The presence of living cells in the melted ice was confirmed by epifluorescence microscopy after staining using the Live/Dead Baclight staining kit (Invitrogen, USA) as previously described (23).

### 16S rRNA gene colony PCR

All colonies that appeared on agar plates after 8 d were transferred to PCR tubes and genomic DNA was extracted by heating in a microwave (600 W, 1 min). DNA was amplified using the KOD-FX Neo kit (TOYOBO) with the primers 27F (5′-AGAG TTTGATCCTGGCTCAG-3′) and 1492R (5′-TACGGTTACCTTG TTACGACTT-3′) (7) following the manufacturer’s procedure. The thermal cycler conditions used for colony PCR were 2 min at 94°C; then 30 cycles of 10 s at 98°C, 30 s at 55°C, and 90 s at 68°C; followed by holding at 4°C. The amplicons were purified using the NucleoSpin Gel and PCR Clean-up kit (Takara, Japan), the expected size and quantity were confirmed by electrophoresis using the 1.5% agarose gel H14 (Takara) and sequenced using the ABI Prism Big Dye Terminator Cycle Sequencing Ready Reaction Kit (Applied Biosystems, USA) according to manufacturers’ protocols. A partial sequence of the 16S rRNA gene was obtained using the 27F primer. Representative OTUs (operational taxonomic units) were chosen from colonies on plates with different rRpf concentrations for almost full-length 16S rRNA gene sequencing using the primers 27F and 1492R, as described above, and 520R (5′-ACCGCGGCKGCTGGC-3′), 357F (5′-CTACGGGAGGCAGCAG-3′), 920F (5′-AAACTCAAAGGAATTGACGG-3′), and 1080R (5′-CCCAACATCTCACGAC-3′). These representative sequences have been deposited into the DNA Databank of Japan under accession numbers AB983161–AB983171.

### 16S rRNA gene sequence analyses

Classification to the genus level and the identification of possible chimera of 16S rRNA gene sequences from all sequenced bacterial colonies were checked using DECIPHER (32). Ambiguous sequence regions were trimmed and sequences were aligned using Clustal Omega (30) to group sequences into OTUs based on 99% sequence identity. The best match to a bacterial type strain at the species level was determined using Sequence Match in the Ribosomal Database Project (RDP, release 11) (2).

### Statistical analysis

All data are presented as means and standard error (±SE). The dosage effect of rRpf was determined using a least square regression analysis of log transformation rRpf concentrations versus the d of the lag phase or growth rates. Correlations were considered significant if *p* <0.05 after 9999 permutations. Significant differences between the numbers of CFUs on plates with different rRpf amounts added were determined using the Student’s *t*-test, with differences being considered significant if *p* <0.05. Statistical analyses were performed using programs available in the PAST software package (5).

## Results

### Purified recombinant Rpf protein produced in *Rhodococcus erythropolis*

**Fig. 1:**
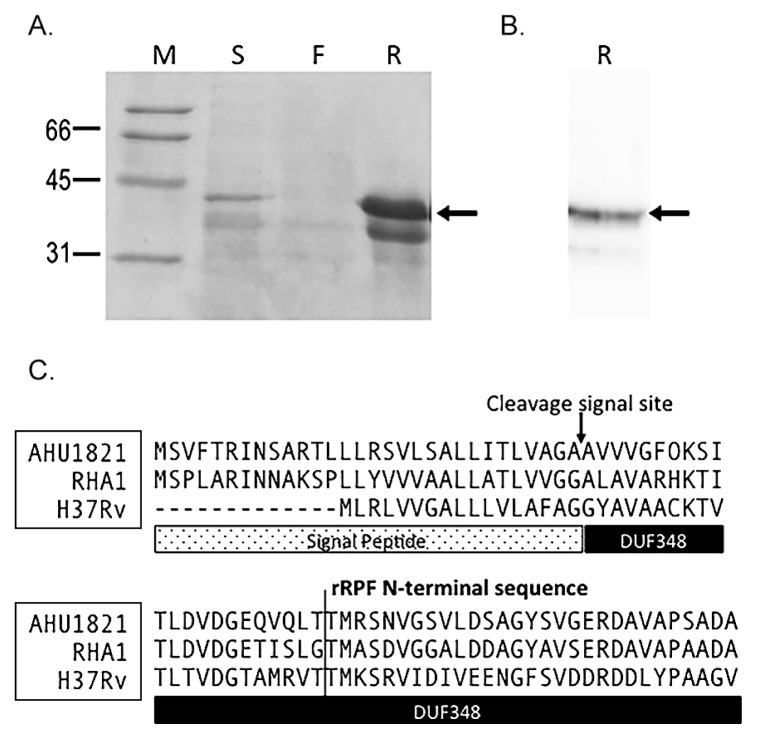
Expression and purification of the histidine-tagged recombinant Rpf (rRpf) protein from *Rhodococcus erythropolis* M1218 confirmed by SDS-PAGE (A) and Western blot hybridization analysis using an anti-His antibody (B). Arrows in panel A, B show the 39 kDa rRpf variant protein. M; molecular weight standard, R; histidine-tagged recombinant Rpf protein fraction, S; culture supernatant, F; flow through the fraction in the purification step. (C) Alignment of the N-terminal sequence of Rpf from *Tomitella biformata* AHU1821, *Rhodococcus jostii* RHA1, and *Mycobacterium tuberculosis* H37Rv with the signal peptide and DUF348 (Rpf conserved domain of unknown funtion) regions highlighted below the sequence. The arrow marks the theoretical signal cleavage site determined using SignalP 4.0 (21) and the line marks the terminal sequence of the two variants of the recombinant Rpf protein.

After purification, the recombinant Rpf from *R. erythropolis (*rRpf-R) produced two bands, estimated to be 32 and 39 kDa in size, on an SDS-PAGE gel (Fig. 1A). Only the 39 kDa band produced a strong signal on a western blot when hybridized with the anti-His antibody (Fig. 1B). A sequence analysis of the N-terminal end of these two bands indicated that both matched the same region of the deduced protein sequence, positions 53–67 of the *T. biformata* rpf gene (Fig. 1C). Using SignalP 4.0 (21), this position was determined to be 22 amino acid residues downstream of the predicted cleavage site based on the deduced Rpf sequence from *T. biformata*. These results indicated that the first 52 amino acids were removed in host cells. The estimated size of the rRpf protein, excluding these 52 amino acids, was 35.8 kDa. The identical N-terminal amino acid sequence of the two variants of rRpf-R suggested that rRpf was expressed in two different forms. Some protein modification may have affected the sensitivity or efficiency of detection.

### Confirmation of recombinant Rpf protein activity

**Fig. 2:**
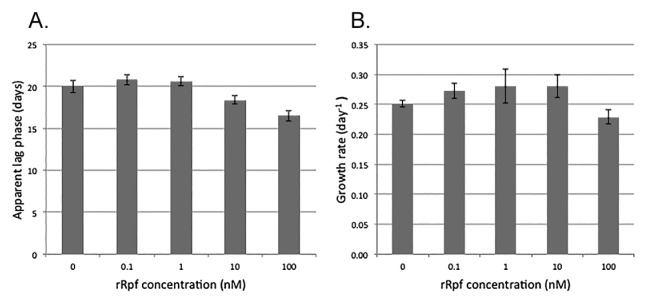
Growth-promoting activity of *Tomitella biformata* recombinant Rpf expressed in *Rhodococcus erythropolis* M1218 on *T. biformata* cultures. (A) Reduction in the lag phase with increases in the rRpf concentration and (B) growth rate (d^−1^). A logarithmic phase culture of *T. biformata* cells was washed, diluted, and added to mMMF medium containing rRpf protein concentrations of 100 nM, 10 nM, 1 nM, 0.1 nM, and 0 nM control. The initial cell concentration of *T. biformata* grown in mMMF medium was 3.10×10^2^ CFU mL^−1^. (*n*=3 per Rpf treatment, error bars represent SE)

**Fig. 3:**
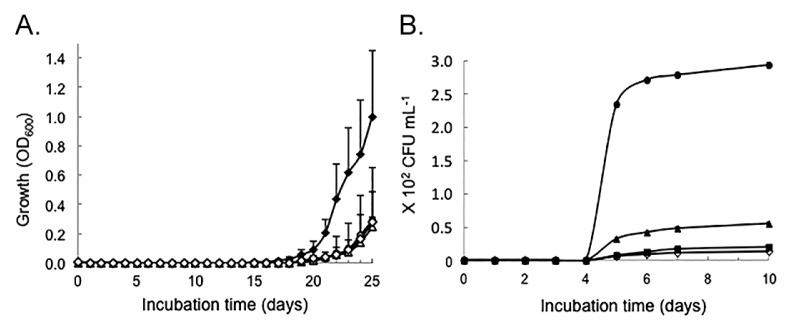
Resuscitation of *Tomitella biformata* in the presence of recombinant Rpf expressed in *Rhodococcus erythropolis* M1218. (A) Non-dividing *T. biformata* cells (37.2 cells mL^−1^) were inoculated into mMMF liquid medium containing final rRpf protein concentrations of 10 nM (○), 1 nM (Δ), 0.1 nM (♦), and 0 nM (⋄) control. (B) *T. biformata (*1.67×10^5^ CFU mL^−2^, 99.9% non-dividing cells) inoculated onto TSBF agar medium containing final rRpf protein concentrations of 300 pM (●), 30 pM (▲), 3 pM (■), and 0 pM (⋄) control. Non-dividing *T. biformata* cells were obtained after a 60-day incubation under oxygen limited conditions. Cells were washed and diluted prior to inoculation into growth media. (*n*=3 per Rpf treatment, error bars represent SE)

The activity of rRpf-R was confirmed using three tests: the growth promotion of dividing cells at low initial concentrations (Fig. 2), the resuscitation of dormant *T. biformata* cells in liquid culture (Fig. 3A), and the resuscitation of dormant *T. biformata* cells on agar medium (Fig. 3B). The apparent lag phase was shortened from 20 d to 16 d (Fig. 2A) in *T. biformata* dividing cells that were grown starting from a low initial cell concentration. Similar results were not obtained for the growth rate (Fig. 2B). A linear regression analysis revealed that a significant decrease occurred in the lag phase (r^2^=0.94, *p*=0.03) with increases in the dosage of rRpf (log transformed). However, a correlation was not observed between growth rates and the rRpf concentrations tested (r^2^=0.47, *p*=0.13). There was also no dosage effect for the resuscitation of non-dividing cells (Fig. 3). The lowest protein concentration tested (0.1 nM) resulted in cultures with significantly (*p* <0.01) higher OD values and a lag phase that was shorter (*p*=0.57) than cultures with higher protein concentrations and the “no protein added” control (Fig. 3A). The resuscitation activity of the rRpf protein was more prominent on solid medium. Colony numbers were approximately 21-fold higher on plates amended with 300 pM rRpf than on control plates (Fig. 3B).

### Colony formation from ice wedge samples dosed with rRpf

**Fig. 4:**
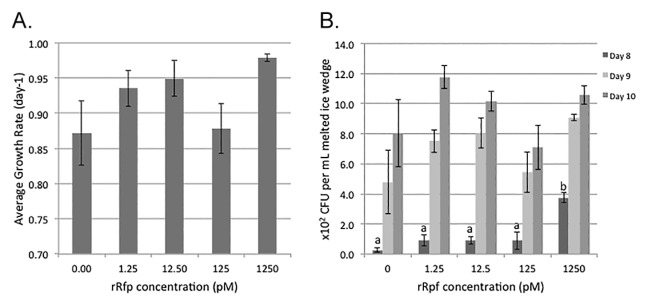
Comparison of (A) growth rates and (B) numbers of colony-forming units (CFU) on R2A agar medium inoculated with the melted ice wedge and different concentrations of recombinant Rpf protein produced using the *Rhodococcus erythropolis* pTip expression vector. CFUs determined from a 10-fold dilution of the melted Fox tunnel ice wedge W4. Plates were incubated at 15°C for 14 days and CFUs counted daily, only days 8–10 are illustrated. (*n*=3 per Rpf treatment, error bars represent SE). Significant differences were only found on day 8, as indicated by letters over the bars (*p* <0.05).

The rRpf dosage effect (r^2^=0.197, *p*=0.39) did not correlate with the growth rate of colonies appearing on plates (Fig. 4A). On the other hand, a significantly (*p* <0.05) greater number of CFUs was observed on plates dosed with 1250 pM of protein than with all other doses tested after 8 d (Fig. 4B). Colony numbers were approximately 14- and 2-fold higher on these plates than on non-amended plates after 8 and 9 d of incubation, respectively. However, after 10 d of incubation, the differences observed in the number of CFUs were no longer significant due to the high variation in cultivable bacterial numbers between experimental replicates.

### 16S rRNA gene identification of colonies formed from ice wedge samples

A total of 192 colonies were collected after 8 d of incubation and their 16S rRNA gene sequences were determined (Table 1). Since significant differences were observed in the number of CFUs after 8 d, this was chosen for sequencing to determine if specific taxa were differentially resuscitated from the ice wedge sample. Due to variations in colony numbers among the Rpf treatments, differences were noted in the number of sequences determined per treatment. A comparison of 124 non-ambiguous sequences (<575 bp) indicated that all represented a single OTU with >99% sequence identity to each other. The closest matching bacterial strain was *Brevibacterium antiquum* strain VKM Ac-2118 (AY243344) with >99% sequence identity. The 11 almost full length sequences that were determined confirmed that the highest identity to a type strain was *B. antiquum* strain VKM Ac-2118 with >98% sequence identity (1387/1413; 4 gaps) and the tentative new species *B. antarcticum* DVS 5a2 (AJ577724) with >98% identity (1397/1427; 6 gaps).

## Discussion

We herein demonstrated that a purified recombinant Rpf protein (rRpf) increased the number of colonies formed from an environmental sample over that on plates with no or low rRpf additions during the earlier stages of incubation. A melted permafrost ice wedge was chosen as the environmental sample because it was the habitat from which *T. biformata*, the source of the *rpf* gene used in these experiments, was first isolated. Although *T. biformata* did not grow from this sample, the colonies were all *Brevibacterium* or a related genus in the phylum *Actinobacteria*. Although only one bacterial species grew from this ice wedge, the number of different taxa, especially *Actinobacteria*, was limited in these samples; therefore, diversity among colonies was not high. The specific activity of Rpf on *Actinobacteria* species was expected because *rpf*-like genes are widely distributed among this phylum (23, 26). Other phyla have been reported to have *rpf*-like genes, but with different sequences and specificities (24). These findings suggest that *T. biformata* Rpf is specific for stimulating the growth of *Actinobacteria*; however, the diversity of the taxa from this group that it can act upon currently remains unknown and requires further investigation.

The main difference between exogenous rRpf treatments on colony formation from the ice wedge sample was when colonies first became evident on the agar plates. This may have occurred because rRpf resuscitated cells from a dormant or non-dividing state and/or promoted the growth of cells once they started to divide. Difficulties have been associated with differentiating the rRpf resuscitation of non-dividing cells from the growth promotion of dividing cells using ice wedge samples because cells maybe in diverse physiological states in the environment and cannot be easily differentiated in natural samples. However, the results of the control experiments using *T. biformata* in dividing and non-dividing states indicated that rRpf mediated both functions. Colony formation occurred sooner in cultures that had been induced into a non-dividing state and in cultures inoculated with low concentrations of dividing cells. This result was supported by the growth and resuscitation-promoting activities of *T. biformata* rRpf expressed in *E. coli*, which we reported previously (23) and as demonstrated by others using rRpf from *Mic. luteus* and *Myc. tuberculosis* (16, 18).

Although the rRpf from *T. biformata* was functional, its activity differed from the phylogenetically closest RpfB in *Myc. tuberculosis*, which has been functionally studied. The *T. biformata* rRpf protein was required at nM concentrations to resuscitate non-dividing cells in liquid cultures, a markedly higher concentration than the pM amounts of rRpf from *Myc. tuberculosis* (18), which was similar to the range reported for *Mic. luteus* (16). Furthermore, the *T. biformata* rRpf protein promoted the growth of cultures of low density-dividing *T. biformata* cells. This was not reported previously for low inoculum *Myc. bovis* cultures dosed with rRpfB from *M. tuberculosis*, although its four other Rpf-like proteins (RpfA, C, D, and E) were shown to promote growth (18). *T. biformata* rRpf (300 pM) also increased colony numbers on agar plates, which had not been reported in earlier studies on *Myc. tuberculosis* (27); however, a later study did show growth on agar plates using a markedly higher concentration, 1 mg of rRpfB (33). Previous studies on rRpf suggested that biological activity was lost when it was added to molten agar or spread on agar surfaces (10, 17, 18). Similar to other studies (16, 18), the concentration of rRpf required for cross species activity using the ice wedge samples differed from the control experiments using non-dividing cells from *T. biformata*. These differences in activity may have been due to differences in the protein sequence and structure of *T. biformata* Rpf from other functionally known Rpf (8) as well as Rpf recognition by non-dividing cells of different bacterial species. This hypothesis warrants more in-depth genetic analyses in the future.

Another factor potentially contributing to differences between these various studies and possibly between replicate experiments using the same rRpf source may be the physiological heterogeneity of cells. Populations of *Vibrio parahaemolyticus* induced into a non-dividing state were found to still contain a subset of dividing cells that started to re-grow when conditions were changed and not because of cell resuscitation (3). This spontaneous regrowth of cells has been referred to as the “scout hypothesis”, the stochastic awakening of dormant cells (1, 4). It currently remains unknown whether cell awakening is truly spontaneous or if some type of cell signaling is a contributing factor. Rpf has been shown to hydrolyze peptidoglycan-producing muropeptide fragments that act as signaling compounds to activate the PknB domain, which plays an important role in regulating cell division and cell wall synthesis (16). If the scout hypothesis is true, then Rpf may be contributing more to growth promotion rather than cell resuscitation. This requires further investigation in order to differentiate the contribution of these two mechanisms to dormant cell resuscitation.

Although the results of the present study indicated that rRpf from *T. biformata* was required at higher concentrations than other sources of Rpf (16, 18), it was difficult to estimate the concentration of biologically active molecules. There were two variants of Rpf proteins produced using this expression system, and either one or both were active. Other studies also reported that Rpf and rRpf existed in different conformations (6, 19). The different conformations of proteins collected from the supernatants of *Corynebacterium glutamicum* and when over-expressed as a recombinant (6) indicates these were not experimental artifacts. However, the contribution of these different protein conformations to Rpf activity and specificity still needs to be elucidated.

## Conclusion

We herein demonstrated that Rpf exhibited cross species activity and increased the colony number of *Actinobacteria* from a permafrost ice wedge, the environment from which *T. biformata* was originally isolated. Rpf appears to be active against other members of the *Actinobacteria*, as suggested previously. Higher concentrations of rRpf from *T. biformata* were needed for growth and resuscitation-promoting activities in the present study than previously reported. These results demonstrate the need for further studies on Rpf from different *Actinobacteria* in order to understand its role and ecological importance in the environment. Future investigations may also develop a method by which to increase the fraction of cells that can be cultivated from environmental samples.

## Figures and Tables

**Table 1:** Number of sequences determined from plates exposed to increasing doses of *Tomitella biformata* recombinant Rpf^a^

Rpf concentration (pM)	Number of colonies sequenced	Number of sequences obtained^a^	Number of non-ambiguous sequences^b^
0	8	8	8
1.25	23	21	6
12.50	28	28	15
125.00	25	25	17
1250.00	108	95	78

Total	192	177	124

a *Tomitella biformata* Rpf cloned into the pTip vector (20) and expressed in *Rhodococcus erythropolis* M1218.

b Sequences may not have been produced if cells were insufficiently lysed

c After removing sequences with any uncalled bases that could not be resolved from the sequence trace files.
